# No Statistically Apparent Difference in Antiviral Effectiveness Observed Among Ribavirin Plus Interferon-Alpha, Lopinavir/Ritonavir Plus Interferon-Alpha, and Ribavirin Plus Lopinavir/Ritonavir Plus Interferon-Alpha in Patients With Mild to Moderate Coronavirus Disease 2019: Results of a Randomized, Open-Labeled Prospective Study

**DOI:** 10.3389/fphar.2020.01071

**Published:** 2020-07-14

**Authors:** Yin-Qiu Huang, Sheng-Quan Tang, Xiao-Lei Xu, Yan-Ming Zeng, Xiao-Qing He, Yao Li, Vijay Harypursat, Yan-Qiu Lu, Yan Wan, Lu Zhang, Qiang-Zhong Sun, Nan-Nan Sun, Gui-Xue Wang, Zhong-Ping Yang, Yao-Kai Chen

**Affiliations:** ^1^ National Key Laboratory for Infectious Diseases Prevention and Treatment with Traditional Chinese Medicine, Chongqing Public Health Medical Center, Chongqing, China; ^2^ Division of Infectious Diseases, Chongqing Public Health Medical Center, Chongqing, China; ^3^ Division of Tuberculosis, Chongqing Public Health Medical Center, Chongqing, China; ^4^ School of Biological Engineering, Chongqing University, Chongqing, China

**Keywords:** ribavirin, interferon-alpha, lopinavir/ritonavir, mild to moderate COVID-19, effectiveness and safety

## Abstract

**Background:**

Currently, severe acute respiratory syndrome coronavirus 2 (SARS-CoV-2) has spread globally, causing an unprecedented pandemic. However, there is no specific antiviral therapy for coronavirus disease 2019 (COVID-19). We conducted a clinical trial to compare the effectiveness of three antiviral treatment regimens in patients with mild to moderate COVID-19.

**Methods:**

This was a single-center, randomized, open-labeled, prospective clinical trial. Eligible patients with mild to moderate COVID-19 were randomized into three groups: ribavirin (RBV) plus interferon-α (IFN-α), lopinavir/ritonavir (LPV/r) plus IFN-α, and RBV plus LPV/r plus IFN-α at a 1:1:1 ratio. Each patient was invited to participate in a 28-d follow-up after initiation of an antiviral regimen. The outcomes include the difference in median interval to SARS-CoV-2 nucleic acid negativity, the proportion of patients with SARS-CoV-2 nucleic acid negativity at day 14, the mortality at day 28, the proportion of patients re-classified as severe cases, and adverse events during the study period.

**Results:**

In total, we enrolled 101 patients in this study. Baseline clinical and laboratory characteristics of patients were comparable among the three groups. In the analysis of intention-to-treat data, the median interval from baseline to SARS-CoV-2 nucleic acid negativity was 12 d in the LPV/r+IFN-α-treated group, as compared with 13 and 15 d in the RBV+IFN-α-treated group and in the RBV+LPV/r+ IFN-α-treated group, respectively (*p*=0.23). The proportion of patients with SARS-CoV-2 nucleic acid negativity in the LPV/r+IFN-α-treated group (61.1%) was higher than the RBV+ IFN-α-treated group (51.5%) and the RBV+LPV/r+IFN-α-treated group (46.9%) at day 14; however, the difference between these groups was calculated to be statistically insignificant. The RBV+LPV/r+IFN-α-treated group developed a significantly higher incidence of gastrointestinal adverse events than the LPV/r+ IFN-α-treated group and the RBV+ IFN-α-treated group.

**Conclusions:**

Our results indicate that there are no significant differences among the three regimens in terms of antiviral effectiveness in patients with mild to moderate COVID-19. Furthermore, the combination of RBV and LPV/r is associated with a significant increase in gastrointestinal adverse events, suggesting that RBV and LPV/r should not be co-administered to COVID-19 patients simultaneously.

**Clinical Trial Registration:**

www.ClinicalTrials.gov, ID: ChiCTR2000029387. Registered on January 28, 2019.

## Introduction

The outbreak of the coronavirus disease 2019 (COVID-19), caused by severe acute respiratory syndrome coronavirus 2 (SARS-CoV-2), has been declared a pandemic by the World Health Organization (WHO) (WHO, 2020). In addition to the dramatic and unprecedented challenges for the entire global community in facing the disease, there remains no specific antiviral treatments for COVID-19 at this time. Thus, efforts to investigate the effectiveness of treatments proposed to combat the etiological agents of similar past viral outbreaks are necessary in order to discover potential direct antiviral therapies for COVID-19, to effectively complement existing supportive care. As described by Zhu et al. (2020), SARS-CoV-2 shares phylogenetic traits with severe acute respiratory syndrome coronavirus (SARS-CoV) and Middle East respiratory syndrome coronavirus (MERS-CoV) despite being genetically distinct. As such, antiviral treatments that were used to target SARS-CoV and MERS-CoV may provide some insight into potential COVID-19 therapies.

Antiviral research during the SARS and MERS outbreaks resulted in the identification of several compounds that may potentially target coronavirus replication directly, or may modulate the immune response to coronavirus infection. For example, a combination of RBV and IFN, has been demonstrated to be effective in reducing MERS-CoV replication *in vitro* (Falzarano et al., 2013a), and has shown a synergistic antiviral effect on MERS-CoV-infected Vero cell lines, which resulted in a consequent decrease in viral protein levels (Chen et al., 2004; Morgenstern et al., 2005). In addition, treatment with IFN and RBV decreased virus replication, moderated host immune responses, and improved clinical outcomes in MERS-CoV-infected rhesus macaques (Falzarano et al., 2013b). In a MERS-CoV study, the survival rate at 14 d after diagnosis was significantly higher in the IFN plus RBV group compared with the control group (70% vs. 29%; *p*=0.004), even though the survival rate did not differ significantly at 28 d (30% vs. 17%; *p*=0.054) (Omrani et al., 2014). The antiviral drug combination of lopinavir/ritonavir (LPV/r) has also been shown to inhibit coronavirus infection in cell cultures at low-micromolar concentrations (de Wilde et al., 2014), and animal experiments have showed that LPV/r combined with IFN effectively reduced virus titers, and induced improvement of pulmonary lesions in MERS-CoV-infected mice (Momattin et al., 2019). Chan et al. (2015) found that the LPV/r plus IFN combination resulted in better clinical scores, decreased weight reduction, less pulmonary infiltrates, and lower viral load in marmosets inoculated with MERS-CoV, compared with controls. Recently, a randomized controlled study observed that the early use of a combination of IFN, LPV/r, and RBV was effective for the treatment of COVID-19, compared with LPV/r alone (Hung et al., 2020).

The available scientific literature suggests that a combination of RBV plus IFN, LPV/r plus IFN, or RBV plus LPV/r plus IFN may be of benefit in patients with COVID-19. The office of National Health Commission of the People’s Republic of China, and the National Administration Bureau of Traditional Chinese Medicine have jointly issued different versions of the “Guidelines for diagnosis and treatment of novel coronavirus pneumonia”, in which LPV/r, IFNα, and RBV are recommended for on-trial use in patients with COVID-19. Based on the Chinese guidelines, and promising results of relevant previous studies, we conducted the present study to compare the efficacy and safety of RBV plus IFN-α, LPV/r plus IFN-α, and RBV plus LPV/r plus IFN-α in patients with mild to moderate COVID-19. We did not set up a blank-controlled or placebo-controlled group out of compassionate and ethical considerations, and the specific limitations to therapeutic variations imposed by national guidelines for COVID-19 in China.

## Methods

### Study Design and Participants

This present study was a single-center, open-labeled, randomized, prospective clinical trial, which included eligible patients diagnosed with mild to moderate COVID-19. This study was approved by the ethics committee of Chongqing Public Health Medical Center (2020-002-01-KY), and was duly registered at the Chinese Clinical Trial Registry (ChiCTR2000029387).

Patients included in our study had to satisfy all the following eligibility criteria: (1) 18–65 years of age; (2) diagnosed as mild to moderate COVID-19; and (3) willing to sign informed consent. We excluded patients if they: (1) were pregnant or breastfeeding women; (2) had aspartate aminotransferase (AST) or alanine aminotransferase (ALT) >5× upper normal limit, creatinine clearance <50 ml/min (Lu and Dong, 2019); (3) were allergic or intolerant to therapeutic drugs; (4) were HIV-positive patients; (5) had severe heart disease, brain disease, lung disease, kidney disease, neoplastic disease, or other systemic diseases, which may have had the potential to influence patients’ adherence to the prescribed antiviral regimens; and (6) withheld informed consent.

Patients meeting all the following criteria were defined to have mild to moderate COVID-19: (1) SARS-CoV-2 nucleic acid testing was positive in the upper respiratory tract *via* nasopharyngeal or oropharyngeal swab samples, or lower respiratory tract *via* expectorated sputum samples, endotracheal aspirate samples, or broncho-alveolar lavage (BAL) samples. (2) Patients were symptomatic with fever, unproductive cough, or dyspnea, and their X-ray or computed tomography scan imaging demonstrated evidence of interstitial pneumonia; (3) respiratory rate (RR)<30 times/min; (4) oxygen saturation (resting-state)>93%; and (5) arterial partial pressure of oxygen (PaO2)/oxygen concentration (FiO2)> 39.9 kPa.

Patients meeting the following criteria were defined to have severe COVID-19: (1) identification of SARS-CoV-2 *via* RT-PCR in nasopharyngeal swab samples, sputum samples, BAL fluid, or blood samples; (2) having at least one of the following clinical conditions: a. respiratory distress (≥30 times/min); b. oxygen saturation ≤93% at rest; c. arterial partial pressure of oxygen (PaO2)/fraction of inspiration O2 (FiO2) ≤39.9 kPa; d. respiratory failure requiring mechanical ventilation; e. septic shock; and f. critical organ failure requiring ICU care.

### Randomization and Masking

All eligible patients were randomly assigned to a random number allocated from a random number sequence generated by a computer, which excluded potential influence from treating physicians, to one of three treatment groups: RBV+IFN-α, LPV/r+IFN-α, and RBV+LPV/r+IFN-α, with an allocation ratio of 1:1:1, and a block size of nine patients each. Laboratory staff performing quantitative or qualitative testing were blinded to treatment allocation, while medical staff were not blinded.

### Data Collection and Quality Assurance

All of the investigators participating in the study were appropriately trained, based on a standard operating procedure (SOP) manual, to ensure patient adherence to the protocol. Efficacy, safety data, and information related to adverse effects were collected at each follow-up visit during the follow-up period. All raw data were recorded in case report forms (CRFs) and in Microsoft Excel (Microsoft Corporation, Redmond, WA, USA). Withdrawals from the study, or missed visits were fully explained on CRFs. Significantly abnormal data, or data that were outside clinically acceptable ranges (laboratory values below or exceeding 20% of the normal range) were required to be explained by the attending physician. We applied this rule for all laboratory values. The study monitor reviewed all CRFs, checked the accuracy of inclusion, exclusion, and withdrawal criteria, as well as ensured that information on the CRFs was in accordance with those in the source electronic medical records.

### Treatment

RBV was given by intravenous injection at a loading dose of 2 g, followed by oral doses of 400–600 mg every 8 h depending on patients’ body weight, for 14 d (Booth et al., 2003; Lu and Dong, 2019). LPV/r was given orally at a dose of 400 mg/100 mg per dose twice per day for 14 d (Cao et al., 2020). IFN-α was given by atomizing inhalation at a dose of 5 million U or 50 mg per dose twice a day for 14 d. All enrolled patients were hospitalized, and medication was dispensed and administered by nurses punctually and in the correct doses, and administered under camera surveillance during hospitalization, so that patient compliance was assured by visual confirmation. Missed doses were administered within 2 h of the prescribed timing. All patients in each cohort could additionally receive nasal cannula oxygen therapy, non-steroidal anti-inflammatory drugs (NSAIDs), oral or intravenous rehydration, electrolyte correction, anti-pyretics, analgesics, and anti-emetic drugs as required by their clinical conditions, as supportive treatment. There were no other differences in treatment administered to individual subjects taking the three different antiviral regimens.

### Study Procedures

Admitted patients with mild and moderate COVID-19 were assessed for eligibility for our trial, and those eligible were asked to sign informed consent before being included in the trial. Then, included patients were randomized to one of the three treatment groups at a ratio of 1:1:1 by means of the computer-generated random allocation schedule. Study visits took place on day 2, day 4, day 7, day 14, and day 28 after initiation of an antiviral regimen. Safety was assessed by interrogation of the participants for development of clinical symptoms, and examination for clinical signs, clinical laboratory tests, and documentation of adverse events. Treatment adherence was assessed by review of clinical diaries that were filled out by our medical staff.

### Outcomes

The primary outcome was the difference in the interval from baseline (initiation of antiviral treatment) to SARS-CoV-2 nucleic acid negativity by nasopharyngeal swab among the three antiviral treatment groups, with each of these two tests at least 24 h apart. The secondary outcomes included the differences among the three groups in the proportion of patients with SARS-CoV-2 nucleic acid negativity at day 14, the mortality rate at day 28, the proportion of patients re-classified as severe cases during the study period, the incidence of adverse events during the study period, and the proportion of therapeutic discontinuations due to adverse events during the study period.

SARS-CoV-2 nucleic acid negativity was defined as the presence of negative SARS-COV-2 results in at least two consecutive nasopharyngeal swabs by reverse transcriptase polymerase chain reaction (RT-PCR), with an interval of at least 24 h between the two time points of swab-taking. Of the two consecutive negative RT-PCR test results, the first negative result was used to calculate the interval between baseline and SARS-COV-2 nucleic acid negativity.

### Statistical Analysis

We applied the mean ( ± SD) to normally distributed data, and median (IQR) to non-normally distributed data. The Kaplan–Meier method was used to estimate median interval to SARS-CoV-2 nucleic acid negativity, and the *p-*value was calculated by log-rank testing. Hazard ratios with 95% confidence intervals were calculated by means of the Cox proportional-hazards model. Categorical variables were analyzed using the chi-squared (χ^2^) test, or the Fisher’s exact test when the expected frequency was less than 5 in one or more cells, and continuous variables were compared using Kruskal-Wallis tests among the three groups. Data were analyzed using SPSS software Version 24 (IBM-SPSS Inc., Chicago, IL, USA). A *p-*value of <0.05 was considered to be statistically significant.

### Role of the Funding Source

The funder of the study had no role in the study design, data collection, data analysis, data interpretation, or writing of the report. The corresponding authors had full access to all the data in the study, and had final responsibility for the decision to submit for publication.

## Results

### Participants and Baseline Characteristics

As a consequence of the novelty of the coronavirus outbreak, no previous data existed in order to calculate an appropriate sample size for our study. Thus, we estimated the sample size according to SARS and MERS data from references, and data from a few discharged COVID-19 patients. We originally planned to recruit a total of 108 subjects because we hypothesized that there would be 2 d difference (standard deviation=1.6) in the primary endpoint among the three cohorts after each receiving one of the three antiviral treatment regimens, based on a power of 80%, and a level of confidence of 95%, while also considering a dropout rate of 10%. However, the efficient control and suppression of the COVID-19 outbreak in Chongqing, due to well-planned and expeditiously executed public health measures in response to the outbreak, resulted in a relative scarcity of appropriate patients to recruit. We therefore finally enrolled 101 participants in total.

**Figure 1:**
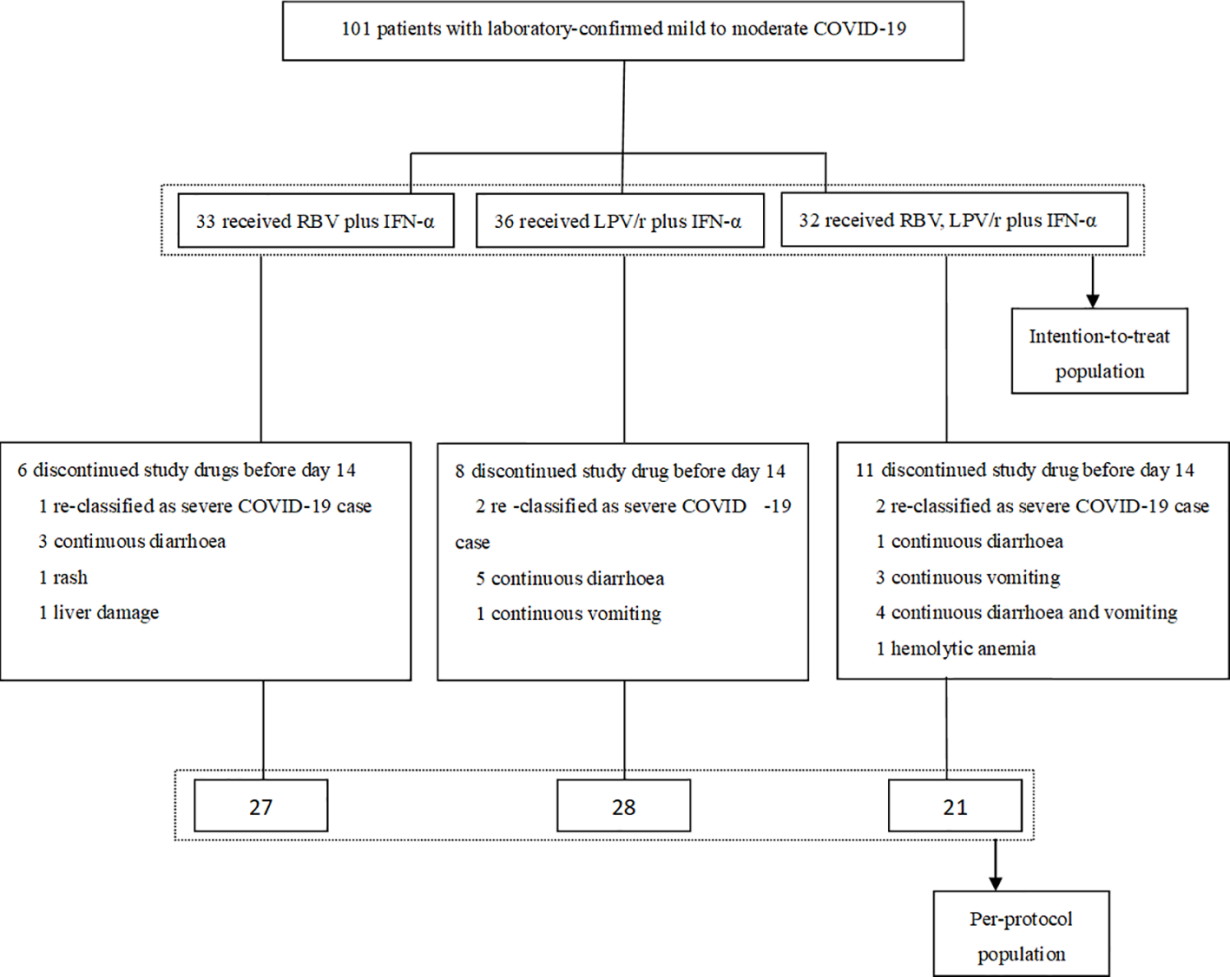
Flow-chart of the study.

All of the 101 participants were enrolled between January 29, 2020 and February 25, 2020, and of these, 33 patients were randomly assigned to the RBV+IFN-α-treated group, 36 were assigned to the LPV/r+IFN-α-treated group, and 32 were assigned to the RBV+LPV/r+IFN-α-treated group. Of the 101 patients, five patients (5.0%) withdrew as a result of re-classification as severe COVID-19 cases, and 20 patients (19.8%) changed their treatment regimens subsequent to adverse events. Finally, 27 (81.8%) patients completed the regimen of RBV+IFN-α, 28 (77.8%) patients completed the regimen of LPV/r+IFN-α, and 21 (65.6%) patients completed the regimen of RBV+LPV/r+IFN-α. A flow diagram detailing the differences in numbers between the intention-to-treat population as compared to the per-protocol (PP) population is shown in **Figure 1**.

The median interval between symptom onset and randomization of the 101 patients in our cohort was 4 d (interquartile range: IQR, 1.5–7.0 d). The mean age of the entire cohort was 42.5 years (SD, 11.5), and 46 (45.5%) of the 101 patients were men. The three most common radiological presentations included ground-glass opacities, consolidation, and pleural thickening. Among all the enrolled patients, 61 (60.3%) had a fever, 45 (44.6%) had a cough, and 29 (28.7%) produced sputum at enrollment. Mean hematological test results on the day of COVID-19 diagnosis were: white blood cell count, 4.8×10^9^/L (IQR, 4.0–6.0×g/L); neutrophil count, 2.9×10^9^/L (2.0–3.9×10^9^/L); hemoglobin, 137×g/L (126–147×g/L); lymphocyte count, 1.5×10^9^/L (1.1–1.9×10^9^/L); platelet count, 182×10^9^/L (141–213×10^9^/L); C-reactive protein, 6.6×mg/L (2.8–14.7×mg/L); erythrocyte sedimentation rate, 24 mm/h (11–47 mm/h); D-dimer, 0.20×mg/ml (0.12–1.78×mg/ml); creatine kinase, 60.1×U/L (44.8–101.0×U/L); CD4^+^ T-cell count, 447×cells/μl (342–648×cells/μl); and CD8^+^ T-cell count, 336×cells/μl (254–465×cells/μl). Baseline demographic and clinical characteristics of patients were comparable among the subjects of the three groups (**Table 1**).

**Table 1:** Baseline characteristics of enrolled patients.

	Total (n=101)	RBV plus IFN-α (n=33)	LPV/r plus IFN-α (n=36)	RBV plus LPV/r plus IFN-α (n=32)	*p* values^*^
**Baseline characteristics**					
Age (years)	42.5 (11.5)	40.3 (12.5)	43.3 (10.4)	43.8 (11.7)	0.41
Men	46 (46%)	18 (55%)	19 (53%)	9 (28%)	0.06
Time from symptom onset to enrollment (days)	4.0 (1.5, 7.0)	4.5 (2.3, 7.0)	3.0 (1.3, 6.8)	4.0 (2.0,7.0)	0.59
Oxygen saturation (%)	97.2 (1.3)	97.2 (1.1)	97.2 (1.4)	97.4 (1.5)	0.59
Respiratory rate (breath/min)	20.0 (19.0, 22.0)	20.0 (19.0, 21.0)	20.0 (19.0, 22.6)	20.0 (18.1, 21.9)	0.29
**Symptom**					
Fever	61 (60.4%)	21 (63.6%)	24 (66.7%)	16 (50%)	0.33
Hypodynamia	15 (14.9%)	5 (15.2%)	9 (25%)	1 (3.1%)	0.03^†^
Dry cough	45 (44.6%)	10 (30.3%)	19 (52.8%)	16 (50%)	0.13
Expectoration	29 (28.7%)	8 (24.2%)	7 (19.4%)	14 (43.8%)	0.07
Diarrhea	10 (9.9%)	5 (15.2%)	1 (2.8%)	4 (12.5%)	0.17^†^
Anorexia	19 (18.8%)	6 (18.2%)	4 (11.1%)	9 (28.1%)	0.20
**Laboratory examination**					
White blood cell (10^9/L)	4.8 (4.0, 6.0)	4.4 (3.6, 5.4)	5.12 (4.0, 5.9)	4.5 (3.9, 5.8)	0.28
Neutrophil (10^9/L)	2.9 (2.0, 3.9)	2.7 (2.0, 3.6)	3.0 (2.4, 3.8)	2.8 (2.0, 4.0)	0.55
Hemoglobin (g/L)	137 (126, 147)	138 (127, 151)	139 (127, 147)	132 (125, 140)	0.62
Lymphocyte (10^9/L)	1.5 (1.1, 1.9)	1.3 (1.0, 1.8)	1.5 (1.2, 1.9)	1.5 (1.2, 1.8)	0.40
Platelet (10^9/L)	182 (141, 213)	149 (137, 204)	198 (150, 217)	178 (144, 209)	0.33
C reactive protein (mg/L)	6.6 (2.8, 14.7)	6.4 (2.2, 13.4)	11.7 (4.1, 28.3)	4.8 (2.3, 10.0)	0.07
Erythrocyte sedimentation rate (mm/h)	24 (11, 47)	22 (9, 54)	28 (21, 50)	27 (13, 47)	0.85
D-Dimer	0.20 (0.12, 0.34)	0.17 (0.09, 0.31)	0.15 (0.11, 0.29)	0.22 (0.14, 0.31)	0.84
Creatine kinase	60.1 (44.8, 101.0)	66.0 (50.5, 110.0)	64 (55.3, 131.8)	54.5 (43.8, 80.0)	0.63
CD4^+^ T-cell count (cells/μl)	487 (342, 648)	448 (315, 605)	506 (260, 674)	503 (397, 653)	0.55
CD8^+^ T-cell count (cells/μl)	336 (254, 465)	322 (276, 465)	349 (209, 459)	348 (271, 475)	0.91
CD4/CD8	1.36 (1.12, 1.78)	1.32 (1.15, 1.76)	1.42 (1.08, 1.71)	1.36 (1.12, 1.78)	0.79
**Chest imaging examination**					
Ground-glass opacity	87 (86.1%)	25 (75.8%)	34 (94.4%)	28 (87.5%)	0.10^†^
Bilateral	72 (71.3%)	20 (60.6%)	27 (75%)	25 (78.1%)	0.25
Symmetry	84 (83.2%)	24 (72.7%)	33 (91.7%)	27 (84.4%)	0.11
Patchy	50 (49.5%)	14 (42.4%)	19 (52.8%)	17 (53.1%)	0.61
Grid-like change	28 (27.7%)	7 (21.2%)	12 (33.3%)	9 (28.1%)	0.53
Nodular	13 (12.9%)	2 (6.1%)	7 (19.4%)	4 (12.5%)	0.28^†^
Pleural effusion	2 (2%)	0 (0%)	1 (2.8%)	1 (3.1%)	0.76^†^
Septal thickening	10 (9.9%)	1 (3%)	4 (11.1%)	5 (15.6%)	0.24^†^
Pleural thickening	43 (42.6%)	14 (42.4%)	15 (41.7%)	14 (43.8%)	0.99

Data are present as n (%), mean ( ± SD) for normally distributed data, or median (IQR) for not- normally distributed data. ^*^p values from χ^2^ tests for categorical variables, the Kruskal-Wallis tests for continuous variables, unless otherwise specified. ^†^ Fisher exact test was used.

RBV, ribavirin; IFN-α, interferon-α; LPV/r, lopinavir/ritonavir.

### Median Interval Between Baseline and SARS-CoV-2 Nucleic Acid Negativity

**Figure 2:**
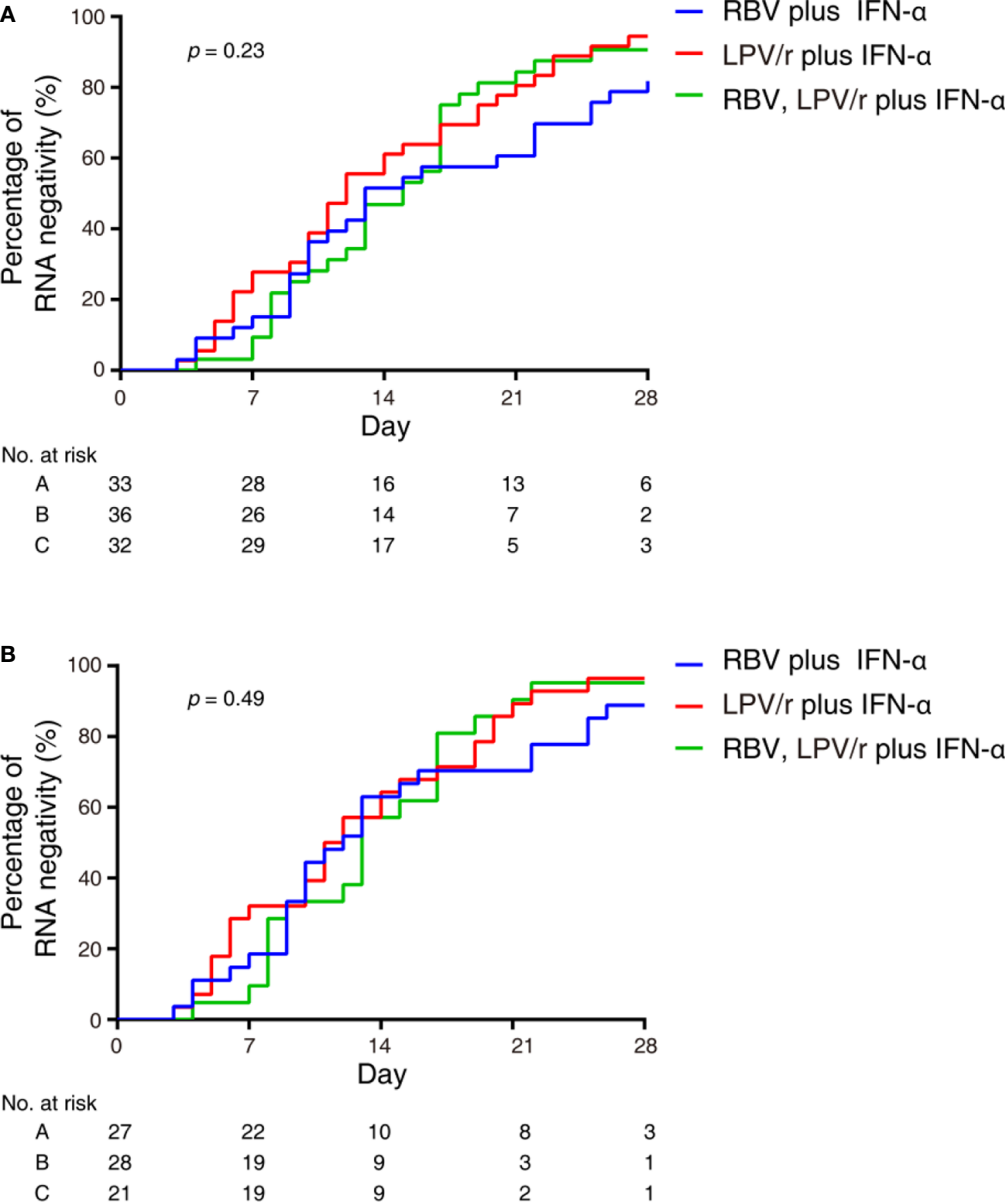
Kaplan–Meier plot depicted in the intention-to-treat population **(A)** and the per-protocol population **(B)**.

In the intention-to-treat (ITT) population, the median intervals from baseline to SARS-CoV-2 nucleic acid negativity was 13, 12, and 15 d in the RBV+IFN-α-treated group, the LPV/r+IFN-α-treated group, and the RBV+LPV/r+IFN-α-treated group, respectively (*p*=0.23). Upon statistical analysis, no significant differences in median interval from baseline to SARS-CoV-2 nucleic acid negativity were found among these groups, and between any two of the three groups. The hazard ratio of nucleic acid negativity in the group taking LPV/r+IFN-α was 1.50 (95% confidence interval [CI], 0.89, 2.52), and the hazard ratio of nucleic acid negativity in the group taking RBV+LPV/r+IFN-α was 1.39 (0.80, 2.41), compared with RBV+ IFN-α (**Table 2** and **Figure 2A**).

**Table 2:** Outcomes in the intention-to-treat population.

	Total (n=101)	RBV plus IFN-α (n=33)	LPV/r plus IFN-α (n=36)	RBV plus LPV/r plus IFN-α (n=32)	*p* values^*^
Fever clearance time (days) ^††^	4 (2.0, 7.0)	4.5 (2.3, 7.8)	4.0 (1.8, 7.0)	3.0 (1.3, 7.8)	0.55
Time of PCR negative (days)	13.0 (9.0, 21.5)	13.0 (9.0, 25.5)	12.0 (7.0, 19.0)	15 (9.3, 17.8)	0.42
CT obviously improvement (days) ^†††^	9.0 (7.5,13.5)	11.0 (8.0, 14.0)	9.0 (6.0, 14.0)	9.0 (7.5, 12.5)	0.76
Hospital stay (days)	17 (12, 24)	17 (12, 28)	15 (10, 24)	18 (13, 22)	0.56
Re-classified severe COVID-19	5 (5.0%)	1 (3.0%)	2 (5.6%)	2 (6.3%)	0.58^†^
Death	0	0	0	0	–

Data are n (%), or median (IQR). CT: Computed Tomography. ^*^p values were calculated by χ2 test or the Kruskal-Wallis test, unless otherwise specified. ^†^ Fisher exact test was used. ^††^Available patients’ data of fever clearance time were 58 (57.4%), 20 (60.0%), 22 (61.1%), and 16 (50.0%) in total, RBV plus IFN-α, LPV/r plus IFN-α, or RBV plus LPV/r plus IFN-α regimen respectively. ^†††^ Available patients’ data of obvious CT improvement was 89 (88.1%), 29 (87.9%), 31 (86.1%), and 29 (90.6%) in total, RBV plus IFN-α, LPV/r plus IFN-α, or RBV plus LPV/r plus IFN-α regimen respectively.

RBV, ribavirin; IFN-α, interferon-α; LPV/r, lopinavir/ritonavir.

In the PP population, the median intervals from baseline to SARS-CoV-2 nucleic acid negativity were 12, 11, and 13 d in the RBV+IFN-α-treated group, the LPV/r+IFN-α-treated group, and the RBV+LPV/r+IFN-α-treated group, respectively (*p*=0.49). Again, no significant differences were observed among the three groups, and between any two of the three groups. The hazard ratio of nucleic acid negativity in the group taking LPV/r+IFN-α was 1.39 (95% CI, 0.77, 2.51), and hazard ratio of nucleic acid negativity in the group taking RBV+LPV/r+IFN-α was 1.22 (0.64, 2.37), compared with taking RBV+ IFN-α (**Table 3** and **Figure 2B**).

**Table 3:** Outcomes in the per-protocol population.

	Total (n=78)	RBV plus IFN-α (n=27)	LPV/r plus IFN-α (n=28)	RBV plus LPV/r plus IFN-α (n=21)	*p* values^*^
Fever clearance time (days) ^††^	4.0 (2.0, 7.0)	4.5 (2.3, 7.8)	4.0 (1.3, 5.8)	3.0 (2.0, 7.3)	0.66
Time of PCR negative (days)	12.0 (8.0, 18.5)	12.0 (9.0, 22.0)	11.0 (6.0, 18.5)	13 (8.0, 17.0)	0.57
CT obviously improvement (days) ^†††^	9.0 (7.0, 12.0)	9.0 (8.0, 13.0)	8.0 (6.0, 12.5)	10.0 (7.0, 12.0)	0.48
Hospital stay (days)	14.5 (11.0, 19.0)	15.0 (11.0, 25.0)	14.0 (9.0, 20.5)	17 (12.5, 19.0)	0.58

Data are n (%), or median (IQR). ^*^p values were calculated by the Kruskal-Wallis test. ^††^ Available patients’ data of fever clearance time were 39 (55.6%), 15 (55.6%), 16 (57.1%), and 8 (28.1%) in total, RBV plus IFN-α, LPV/r plus IFN-α, or RBV plus LPV/r plus IFN-α regimen respectively. ^†††^ Available patients’ data of obvious CT improvement was 67 (88.2%), 23 (85.2%), 25 (89.3%), and 19 (90.5%) in the total, RBV plus IFN-α, LPV/r plus IFN-α, or RBV plus LPV/r plus IFN-α regimen respectively.

RBV, ribavirin; IFN-α, interferon-α; LPV/r, lopinavir/ritonavir; CT, computed tomography.

### Proportion of Patient With SARS-CoV-2 Nucleic Acid Negativity

**Figure 3:**
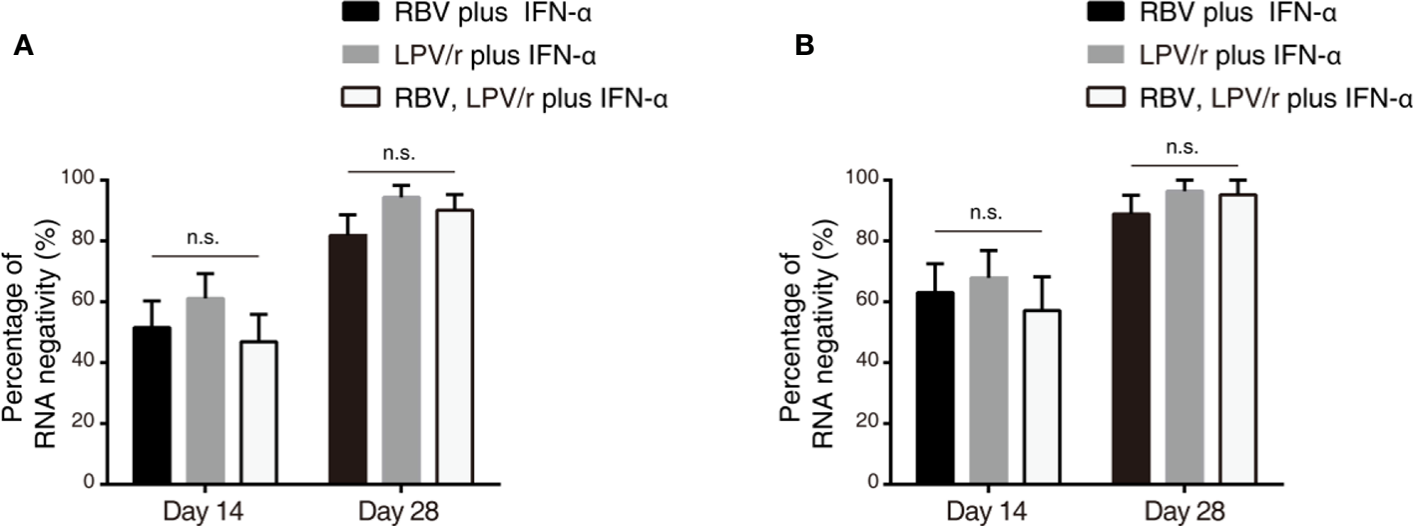
Percentage of severe acute respiratory syndrome coronavirus 2 nucleic acid negativity in the intention-to-treat population **(A)** and per-protocol population **(B)**. The error bars show standard error of mean (SEM), n.s, not significant.

In the ITT population, the proportion of patients with SARS-CoV-2 nucleic acid negativity at day 14 in the RBV+IFN-α-treated group, the LPV/r+IFN-α-treated group and the RBV+LPV/r+IFN-α-treated group were 51.5% (17/33), 61.1% (22/36), and 46.9% (15/32) respectively, and 81.8% (27/33), 94.4% (34/36), and 90.1% (29/32) respectively, at day 28 (**Figure 3A**). However, the differences were statistically insignificant among the three groups, and between any two of the three groups.

In the PP population, the proportion of patients with SARS-CoV-2 nucleic acid negativity at day 14 in the RBV+IFN-α-treated group, the LPV/r+IFN-α-treated group, and the RBV+LPV/r+IFN-α- treated group were 63.0% (17/27), 67.9% (19/28), and 57.1% (12/21) respectively, and 88.9% (24/27), 96.4% (27/28), and 95.2% (20/21) respectively at day 28, as shown in **Figure 3B**. No significant differences were found among the three groups, and between any two of the three treatment groups.

### Proportion of Patients With Illness Progression

We also assessed proportion of patients with illness progression among the three groups by comparing different proportion of patients re-classified as severe cases, and mortality rate during the study period. We found that one patient (3.0%) in the RBV+IFN-α-treated group, two patients (5.6%) in the LPV/r+IFN-α-treated group, and two patients (6.3%) in the RBV+LPV/r+IFN-α-treated group had been re-classified as severe cases of COVID-19. However, no statistically significant difference was observed among the three groups, and between any two of the three groups. Gratifyingly, there was no mortality in our cohort of 101 patients during the study period (**Table 2**).

### Safety Evaluation

**Figure 4:**
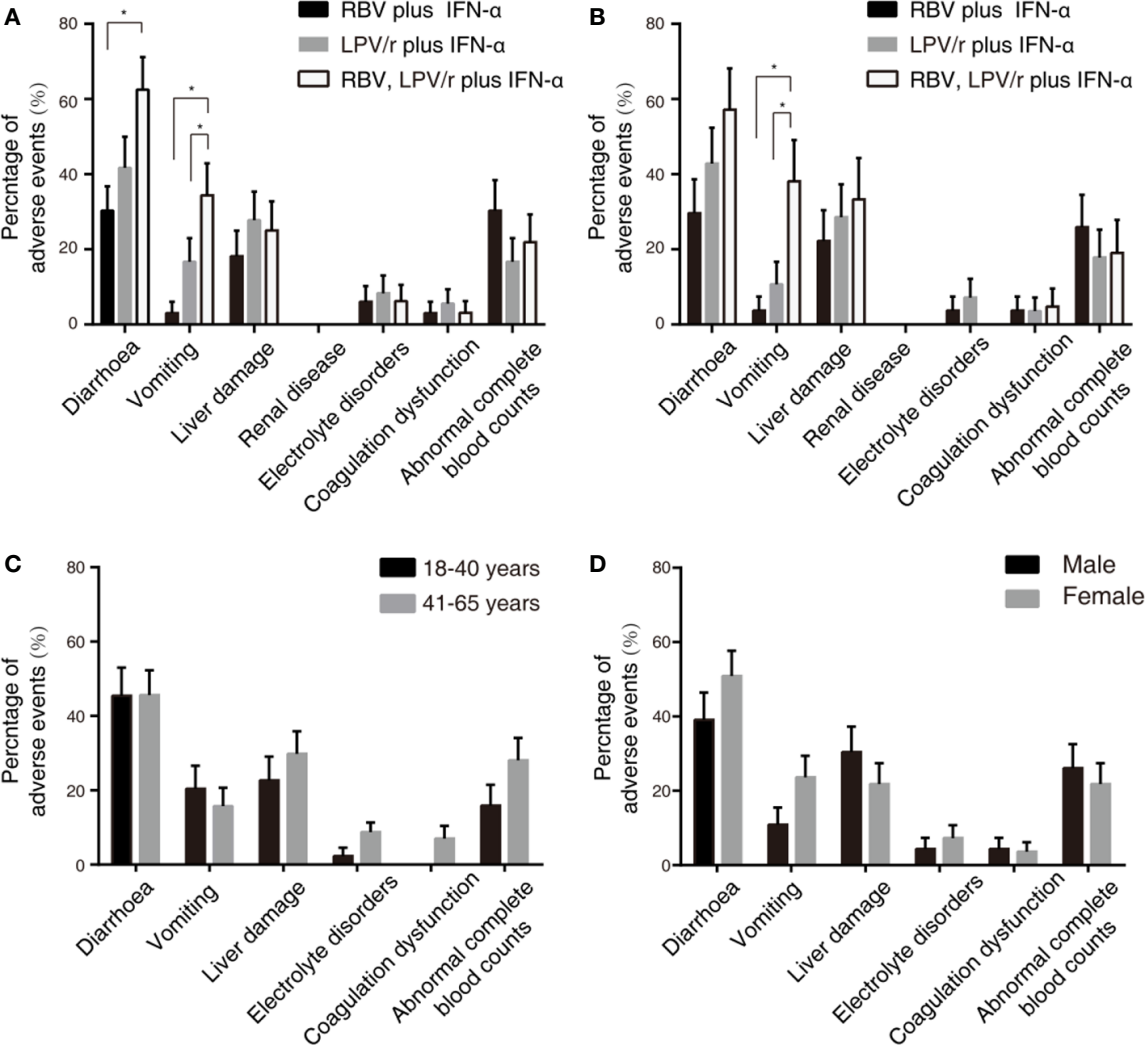
Percentages of adverse events in the intention-to-treat (ITT) population **(A)** and the per-protocol population **(B)** among the three different therapeutic regimens, and stratified by ages **(C)** or by gender **(D)** in the ITT population. The error bars represent standard errors of mean (SEM). Comparisons with an asterisk (*) indicate statistical significance (*p* < 0.05), otherwise indicate no significance.

A total of 23 patients (69.7%) in the RBV+IFN-α-treated group, 26 patients (72.2%) in the LPV/r+IFN-α-treated group, and 30 patients (93.8%) in the RBV+LPV/r+IFN-α-treated group reported adverse events during study period (**Table 4**). A significantly higher incidence of diarrhea and vomiting was observed in the RBV+LPV/r+IFN-α-treated group compared to the other two groups, in the ITT population (**Table 4** and **Figure 4A**). However, a significantly higher proportion of vomiting only was observed in the RBV+LPV/r+IFN-α-treated group, compared with the other two groups in the PP population (**Figure 4B**). There were no significant differences in the incidence of other adverse events among the three groups, including liver damage, electrolyte disorders, coagulation dysfunction, and abnormal complete blood counts (**Figures 4A, B**). In addition, no patients appeared to develop renal dysfunction in our study. We found that no statistically significant differences existed between incidence of adverse events either in age stratification (**Figure 4C**), or gender (**Figure 4D**) in the ITT and the PP populations (data not shown). No serious adverse events occurred during the course of the present study.

**Table 4:** Adverse events in the study population.

	RBV plus IFN-α (n=33)	LPV/r plus IFN-α (n=36)	RBV plus LPV/r plus IFN-α (n=32)	*p* value
**Adverse events of grade 1 or 2**	23 (69.7%)	26 (72.7%)	29 (90.6%)	0.09^*^
Diarrhea	7 (21.2%)	14 (38.9%)	19 (59.4%)	<0.01^*^
Vomiting	1 (3.03%)	6 (16.7%)	11 (34.4%)	<0.01^*^
Electrolyte disorders	2 (6.7%)	3 (8.3%)	2 (6.3%)	1.00
Coagulation dysfunction	1 (3.0%)	2 (5.6%)	1 (3.1%)	1.00
Sleep disorders and disturbances	1 (3.0%)	2 (5.6%)	3 (9.4%)	0.60
Leukopenia	7 (21.2%)	6 (16.7%)	6 (18.8%)	0.95
Thrombocytopenia	3 (9.1%)	1 (2.8%)	2 (6.3%)	0.51
Increased total bilirubin	2 (6.1%)	6 (16.7%)	5 (15.6%)	0.37
Increased alanine aminotransferase	4 (12.1%)	1 (2.8%)	1 (3.1%)	0.31
Increased aspartic transaminase	4 (12.1%)	2 (5.6%)	1 (3.1%)	0.39
Hyperlipidemia	0 (0.0%)	1 (2.8%)	0 (0.0%)	1.00
Rash	3 (9.0%)	0 (0.0%)	2 (6.3%)	0.19
Hemolytic anemia	0 (0.0%)	0 (0.0%)	1 (3.1%)	0.32
**Adverse events of grade 3 or 4**	4 (12.1%)	3 (8.3%)	3 (9.4%)	0.92
Diarrhea	3 (9.1%)	1 (2.8%)	1 (3.1%)	0.52
Increased total bilirubin	1 (3.0%)	2 (5.6%)	2 (6.3%)	0.87
Increased alanine aminotransferase	0 (0.0%)	1 (2.8%)	0 (0.0%)	1.00
**Serious adverse events**	0 (0.0%)	0 (0.0%)	0 (0.0%)	–
**Adherence**	27(81.8%)	28(77.8%)	21(65.6%)	0.29^*^

Data are n (%). Asterisk (*) were calculated by χ2 test, otherwise were calculated by Fisher exact test.

RBV, ribavirin; IFN-α, interferon-α; LPV/r, lopinavir/ritonavir.

## Discussion

The current SARS-CoV-2 epidemic is the third serious outbreak of a coronavirus-related disease this century, following the SARS outbreak in China in 2003, and the MERS outbreak in the Middle East in 2012. Unfortunately, this outbreak has evolved into a widespread pandemic causing global apprehension, and the number of patients infected with SARS-CoV-2 is growing exponentially by tens of thousands daily, with hundreds of patients dying every day worldwide. It is therefore of urgent importance to explore viable options for effective antiviral treatment for COVID-19 (Zeng et al., 2020).

In the present randomized trial, results showed that the median intervals between baseline and SARS-CoV-2 nucleic acid negativity in the RBV+IFN-α-treated group, the LPV/r+IFN-α-treated group, and the RBV+LPV/r+IFN-α-treated group were 13, 12, and 15 d in the ITT cohort, and 12, 11, and 13 d in the PP cohort, respectively. There was no significant difference in terms of the primary outcome among the three groups, and between any two of the three groups. In addition, we did not observe any significant difference in the proportion of patients with SARS-CoV-2 nucleic acid negativity among the three groups both at day 14 and at day 28. Nor did we observe any significant difference in proportion of patients with illness progression during the study period among the three groups. These results clearly indicate that there was no noticeable difference among the three therapeutic antiviral regimens in terms of antiviral efficacy when used in patients with mild to moderate COVID-19. We thus concluded that three different combinations of antiviral drugs have the same or similar antiviral efficacy on COVID-19. We did not conduct a subgroup analysis by sex and age for the three antiviral regimens, as the small sample size in our study did not effectively support such sub-set analyses.

During the SARS and MERS outbreaks, injectable IFNα was found to be potentially beneficial in the treatment of patients with SARS and MERS. Atomized IFNα is now being used for the first time to treat coronavirus-infected patients during the COVID-19 outbreak. Detailed pharmacokinetics for atomized IFNα are not completely understood at this stage. The diameter of atomized drug particles determines whether the atomized drug will be delivered to the target site, thus directly affecting the efficacy of the drug. The diameter of atomized particles that can be deposited in the airway and lungs should ideally be between 0.5 and 10 μm, and a particle diameter of 3–5 μm is considered optimal. Wang et al. (2019) found that after injectable IFNα is atomized, the droplet size distribution is suitable for drug delivery in to respiratory tract, and may be deposited in bronchi, bronchioles and lung parenchyma at all levels. After atomization, about 81.3% of IFNα injection particles are between 1 and 5 μm in diameter. Another study showed that the biological retention rate of IFNα was about 96%, and IFNα distribution was found in lung tissue after 2 h (Liu et al., 2013; Wang et al., 2017). Therefore, aerosolized inhalation of IFNα is able to reach the lung parenchymal tissue in order to exert potential beneficial biological effects. IFNα administered by atomizing inhalation could potentially have a prolonged duration of action, and thus may require a reduction of frequency of administration. When the blood and tissue concentrations of IFNα exceeds 10 U/ml, immune cells are stimulated to display antiviral activity within 5 min (Tong, 1994). Falzarano et al. (2013a) found that RBV combined with IFNα may inhibit the replication of MERS-COV in cultured cells at a low therapeutic concentration (250 U/ml), and reduced cytopathic effects (CPE). An animal study observed that the maximum blood concentration (Tmax) of IFNα when given by atomizing inhalation occurred 1 h later than IFNα administered by intramuscular injection. The mean residence time (MRT) was significantly prolonged compared with intramuscular injection, *viz.* 7 h as compared to 16 h. The elimination half-life of IFNα was extended from 7.3 to 12.1 h when given by atomizing inhalation as opposed to injection (Yang et al., 2015). Based on these previous studies, and the national guidelines of China, the doses of antiviral drugs administered in our study were adequate and appropriate.

There are two possible explanations for our findings: first, that the three regimens have very similar or almost identical antiviral efficacy and therefore, no difference in effectiveness could be observed in the analyzed data; and second, that the three regimens do not have any significant antiviral efficacy clinically and therefore, no difference in effectiveness could be observed in the analyzed data. Our research group tends to accept the latter explanation based on the following reasoning: (1) the possibility of three different combinations of drugs having the same or similar antiviral efficacy is exceedingly slim; (2) a recent placebo-controlled clinical trial has shown that LPV/r does not have antiviral efficacy in COVID-19 patients (Cao et al., 2020); (3) a retrospective study has shown that patients receiving potential antiviral drugs such as LPV/r, IFNα, or arbidol had similar viral clearance times when compared to those who did not receive any antiviral drugs (Ling et al., 2020); (4) a perspective study had shown that early combination of IFN, LPV/r, and RBV was effective in suppressing the shedding of SARS-CoV-2, not just in a nasopharyngeal swab, but in all clinical specimens (Hung et al., 2020). However, in our study, we did not set up blank-controlled, or placebo-controlled treatment group out of compassionate and ethical considerations, and the specific limitations to therapeutic variations imposed by national guidelines for COVID 19, and also because of the relative paucity of eligible patients to enroll in Chongqing. Therefore, we cannot definitively rule out the possibility that the three chosen different antiviral drug combinations in our study have identical or very similar antiviral efficacy profiles against SARS-CoV-2.

The adverse events of LPV/r and RBV have been widely reported. Diarrhea and emesis are well-recognized adverse effects of LPV/r therapy, and has been noted previously in studies investigating the role of LPV/r in the treatment of HIV-patients (Cao et al., 2020). Our results showed that in the ITT population, the incidence of diarrhea and vomiting in the RBV+LPV/r+IFN-α-treated group was significantly higher than that of the RBV+IFN-α-treated group, and that of the LPV/r+IFN-α-treated group. In the PP population, a significantly higher proportion of vomiting was observed in the RBV+LPV/r+IFN-α-treated group. IFN does not typically cause gastrointestinal adverse effects, and is especially not expected when IFN is administered by atomizing inhalation. Therefore, the significantly higher increase in the incidence of diarrhea and vomiting in the RBV+LPV/r+IFN-α-treated group may credibly be attributed to the combination of LPV/r plus RBV. As such, we advise that the therapeutic combination of LPV/r and RBV should probably not be prescribed for the management of COVID-19.

Among SARS patients, 58% had hypocalcaemia, 61% had hemolytic anemia, and 46% had hypomagnesaemia (Knowles et al., 2003). We failed to identify any subjects with hypomagnesaemia in our cohort. We observed that the RBV+IFN-α-treated group had the highest degree of abnormal complete blood counts, compared with the RBV+LPV/r+IFN-α-treated group and the LPV/r+IFN-α-treated group. However, there was an absence of statistically calculated significance for the development of abnormal complete blood counts among the three groups.

There are limitations to our study. Firstly, the open-labeled design may have led to bias in the assessment of adverse events and clinical resolution. Nevertheless, centralization of randomization, and strict inclusion and exclusion criteria, are expected to have reduced this possibility. Secondly, the study was not blinded, and it is possible that knowledge of treatment assignment by patients and treating physicians may have influenced clinical outcomes. Thirdly, this was a single-center trial with a limited number of participants in three arms, and thus, the results obtained at our center may not be representative of other hospitals in China. Fourthly, we would have been able to better substantiate the effectiveness and safety of the different antiviral treatment regimens if control groups receiving placebo, or supplement treatment only, had been incorporated into our study design. Fifthly, our small study cohort contributed to an underpowering of our study. Withdrawals as a result of adverse events further contributed to this underpowering. Consequently, further studies of these antiviral drug combinations with larger study cohorts are warranted in order to validate our results. Finally, we did not perform pharmacokinetic measurements of the study drugs, and thus cannot comment on their precise bioavailability in COVID-19 patients.

In conclusion, our results demonstrated that there was no statistically apparent difference among the three antiviral therapeutic regimens in terms of antiviral effectiveness in patients with mild to moderate COVID-19. This could imply that the antiviral effectiveness of LPV/r+IFN-α, RBV+IFN-α, and RBV+LPV/r+IFN-α for the management of COVID-19 is similar. Further, the combination of RBV and LPV/r is associated with a significant increase in gastrointestinal adverse events, suggesting that RBV and LPV/r should not be simultaneously administered to COVID-19 patients for the management of SARS-CoV-2 infection. Although we recognize that our study is underpowered with a small patient cohort, we hope that these early findings may inform future effective management of COVID-19.

## Data Availability Statement

The original contributions presented in the study are included in the article/supplementary material, further inquiries can be directed to the corresponding authors.

## Ethics Statement

The studies involving human participants were reviewed and approved by the ethics committee of Chongqing Public Health Medical Center (2020-002-01-KY). The patients/participants provided their written informed consent to participate in this study.

## Author Contributions

Y-KC and Z-PY conceived the study. LZ, Y-MZ, Q-ZS, N-NS, and YW collected data. S-QT, YL, G-XW, and X-LX analyzed data. Y-QH, S-QT, and Y-KC interpreted the results. Y-QH and S-QT wrote the manuscript. VH, X-QH, and Y-QL edited and revised the article.

## Conflict of Interest

The authors declare that the research was conducted in the absence of any commercial or financial relationships that could be construed as potential conflicts of interest.
